# Activation of G_s_ Signaling in Cortical Astrocytes Does Not Influence Formation of a Persistent Contextual Memory Engram

**DOI:** 10.1523/ENEURO.0056-24.2024

**Published:** 2024-06-14

**Authors:** Aline Mak, Adlin Abramian, Stan L. W. Driessens, Cristina Boers-Escuder, Rolinka J. van der Loo, August B. Smit, Michel C. van den Oever, Mark H. G. Verheijen

**Affiliations:** Department of Molecular and Cellular Neurobiology, Center for Neurogenomics and Cognitive Research, Amsterdam Neuroscience, Vrije Universiteit Amsterdam, Amsterdam 1081 HV, The Netherlands

## Abstract

Formation and retrieval of remote contextual memory depends on cortical engram neurons that are defined during learning. Manipulation of astrocytic G_q_ and G_i_ associated G-protein coupled receptor (GPCR) signaling has been shown to affect memory processing, but little is known about the role of cortical astrocytic G_s_-GPCR signaling in remote memory acquisition and the functioning of cortical engram neurons. We assessed this by chemogenetic manipulation of astrocytes in the medial prefrontal cortex (mPFC) of male mice, during either encoding or consolidation of a contextual fear memory, while simultaneously labeling cortical engram neurons. We found that stimulation of astrocytic G_s_ signaling during memory encoding and consolidation did not alter remote memory expression. In line with this, the size of the mPFC engram population and the recall-induced reactivation of these neurons was unaffected. Hence, our data indicate that activation of G_s_-GPCR signaling in cortical astrocytes is not sufficient to alter memory performance and functioning of cortical engram neurons.

## Significance Statement

Astrocytes structurally and functionally interact with neurons at the synapse, and ample studies demonstrate that astrocytic GPCRs play a role in memory processing. For instance, chemogenetic activation of GPCR-mediated G_i_ or G_q_ signaling in hippocampal astrocytes affects memory formation. However, the involvement of astrocytic G_s_ signaling in formation of a persistent memory, which depends on cortical neurons, is not known. Here, we show that chemogenetic activation of G_s_ signaling in cortical astrocytes has no effect on the encoding or consolidation of persistent fear memory and functionality of memory encoding cortical neurons. Together with previous studies, this indicates that astrocytic GPCR-mediated effects on memory function depend on the G-protein type, brain region, and/or phase of memory processing.

## Introduction

Ample studies have demonstrated that the so-called engram neurons form the neurobiological substrate of a given memory (Josselyn et al., 2015; Tonegawa et al., 2015). These engram cells are sparsely distributed neurons that are activated by learning (Reijmers et al., 2007; Liu et al., 2012) and undergo physiological, structural, and molecular changes to consolidate the newly formed memory (Cruz et al., 2013; Ryan et al., 2015; Rao-Ruiz et al., 2019). Reactivation of engram neurons upon exposure to reminder cues is required for adequate memory recall (Garner et al., 2012; Liu et al., 2012; Denny et al., 2014). Whereas newly formed memories depend on engagement of hippocampal engram neurons (Liu et al., 2012; Denny et al., 2014; Tanaka et al., 2014), memory persistence depends on progressive engagement of engram neurons in cortical areas, in particular the medial prefrontal cortex (mPFC; Kitamura et al., 2017; Matos et al., 2019). This gradual shift to dependence on cortical engram neurons is thought to be established through the process of systems consolidation (Frankland and Bontempi, 2005; Rao-Ruiz et al., 2021).

Astrocytes have a dynamic structural and functional interaction with neurons at the synapse (Badia-Soteras et al., 2020). The wide variety of receptors expressed by astrocytes, including GPCRs, can be activated by neurotransmitters and other small molecules, thereby enabling detection of changes in synaptic activity and other extracellular stimuli (Roux and Cottrell, 2014; Bazargani and Attwell, 2016; Durkee and Araque, 2019; Kofuji and Araque, 2020). GPCRs are membrane bound metabotropic receptors that, upon activation, are known to stimulate several cellular signaling pathways depending on their coupling to G_q_, G_i_, or G_s_ protein complexes (S. H. Lee et al., 2023). Development of Designer Receptors Exclusively Activated by Designer Drugs (DREADDs) has offered the possibility to activate a specific GPCR subtype exclusively in astrocytes, providing important insight into the role of astrocytes in memory function (S. H. Lee et al., 2023). For instance, astrocytic G_q_-DREADD activation in the hippocampal CA1 during contextual fear conditioning (CFC) induces NMDA-dependent long-term potentiation (LTP) in hippocampal neurons and enhances recent fear memory recall, whereas G_i_-DREADD activation during conditioning impairs remote memory retrieval without affecting recent memory, likely by altering neuronal communication between CA1 and the anterior cingulate cortex (Adamsky et al., 2018; Kol et al., 2020). Furthermore, DREADD-mediated G_i_ pathway activation in hippocampal astrocytes lowers LTP threshold and enhances recent memory recall in a conditioned place paradigm (Nam et al., 2019). Together, these studies provide compelling evidence for the involvement of the astrocytic G_q_ and G_i_ pathways in memory function through changes in neuronal activation. However, the role of the astrocytic G_s_ signaling pathway in learning and memory is less well characterized (S. H. Lee et al., 2023).

Activation of endogenous G_s_-coupled receptors in astrocytes induces cAMP production (Vardjan et al., 2014; Oe et al., 2020; S. H. Lee et al., 2023). Conflicting effects have been reported of manipulation of astrocytic G_s_-coupled receptors and cAMP levels on memory function. For instance, activation of G_s_-coupled β_2_-adrenergic receptors during CFC induces the release of lactate in the hippocampus, which is essential for long-term memory consolidation (Gao et al., 2016). Furthermore, elevating cAMP in hippocampal astrocytes by optogenetic stimulation enhances memory retention in an object recognition task (Zhou et al., 2021). The effect of elevated cAMP levels on memory are thought to be mediated by increased glycogen breakdown and lactate shuttling between astrocytes and neurons. Blocking astrocytic lactate transporters impairs recent memory expression (Suzuki et al., 2011; Oe et al., 2020; Zhou et al., 2021). Lactate shuttling induces NMDA receptor-dependent plasticity, LTP, and the expression of immediate early genes like Fos and Arc in neurons (Suzuki et al., 2011; Yang et al., 2014; Zhou et al., 2021). In contrast, brain-wide astrocytic ablation of the G_s_-coupled adenosine A_2A_ receptor enhances spatial memory retrieval, whereas brain-wide chemogenetic stimulation of G_s_ signaling via a modified G_s_-coupled receptor (Rs1) impairs memory consolidation (Orr et al., 2015).

These findings point to an important role of astrocytic G_s_ signaling in memory function, likely depending on the brain region and timing of manipulation. Moreover, previous studies that assessed GPCR signaling in astrocytes focused mainly on the hippocampus and recently acquired memories. Therefore, we aimed to investigate the role of the G_s_ pathway in cortical astrocytes in the formation of a persistent fear memory and corresponding engram neurons in the mPFC that support remote fear memory. We found that chemogenetic activation of astrocyctic G_s_ signaling during encoding and consolidation did not interfere with subsequent expression of remote fear memory, nor with the size and reactivation of the cortical engram cell population.

## Materials and Methods

### Animals

Male double heterozygous TRAP2-tdTomato mice [Fos^2A-iCreER/+^ (Jackson Laboratory stock #030323) crossed with R26^AI14+^ (Jackson Laboratory stock #007914); DeNardo et al., 2019] were 2–3 months old at the start of experiments and individually housed on a 12 h light/dark cycle with food and water available *ad libitum*. Mice were randomly assigned to experimental groups. Behavioral experiments were performed during the beginning of the light phase. All experimental procedures were approved by The Netherlands Central Committee for Animal Experiments (CCD) and the Animal Ethical Care Committee (DEC; AVD1120020174287).

### Constructs

The pAAV-GFAP::HA-rM3Ds-EGFP plasmid was generated from pAAV-GFAP::HA-rM3Ds-IRES-mCitrine (gift from Bryan Roth, Addgene plasmid #50472) by replacing IRES-mCitrine with EGFP from pAAV-GFAP-EGFP (gift from Bryan Roth lab, Addgene plasmid #50473).

### AAV vectors and stereotactic microinjections

AAV-GFAP::HA-rM3Ds-EGFP (1.74 × 10^12^ TU/ml) and AAV-GFAP::EGFP (8.4 × 10^12^ TU/ml) were packaged as serotype 2/5 virus. For stereotaxic microinjections in the mPFC (Van den Oever et al., 2013; Matos et al., 2019), mice received carprofen (0.067 mg/ml, Rimadyl Cattle, Zoetis) in their drinking water starting 24 h before surgery and until 2–3 d after surgery. In addition, an injection of Temgesic (0.05 mg/kg, s.c., RB Pharmaceuticals) was provided 30 min before surgery, and mice were then anesthetized with isoflurane and mounted in a stereotactic frame. Lidocaine (2%, Sigma-Aldrich Chemie) was topically applied to the skull to provide local analgesia. Using a microinjection glass needle, 0.5 µl of AAV-GFAP::HA-rM3Ds-EGFP or AAV-GFAP-EGFP was bilaterally injected in the mPFC (+1.8 mm AP; ±0.45 mm ML; −2.1 mm DV; relative to bregma) with a flow rate of 0.1 µl per min. The mice were kept in individually ventilated cages for a week after surgery and moved to their conventional home-cage for 3 weeks until the start of behavioral experiments.

### CFC

Mice were handled for 2 consecutive days to habituate the animals to the experimenter. Three days later, CFC (Matos et al., 2019) was performed in a Plexiglas chamber with stainless-steel grid floor inside a soundproof cabinet with continuous white noise (68 dB; Ugo Basile). Between each trial, the chamber was cleaned with 70% ethanol. Mice were allowed to explore the CFC context for 180 s prior to the onset of a footshock (0.7 mA, 2 s). After 30 s, mice were returned to their home-cage. During the retrieval session 4 weeks later, the mice were placed back in the context for 180 s. Freezing behavior was analyzed by video tracking using EthoVision XT (Noldus). Freezing bouts were defined as a lack of movement except respiration for at least 1.5 s.

### 4-Hydroxytamoxifen treatment

4-Hydroxytamoxifen (4TM; HB6040, Hello Bio) was injected as aqueous solution (Ye et al., 2016; Matos et al., 2019). First, 4TM was dissolved in DMSO (D8418, Sigma-Aldrich Chemie). This solution was then diluted in saline containing 2% Tween 80 (P1754, Sigma-Aldrich Chemie), followed by the addition of saline. The final solution contained 2.5 mg/ml 4TM, 5% DMSO, and 1% Tween 80 in saline. Animals received 4TM (50 mg/kg, i.p.) 1 h after the CFC training.

### Chemogenetic intervention

For the activation of rM3Ds during encoding, clozapine *N*-oxide (CNO, HB6149, Hello Bio) was dissolved in sterile saline, and mice received 5 mg/kg (i.p.) CNO 30 min before the CFC training session. For the activation of rM3Ds during the consolidation phase, mice received CNO directly after the CFC training session by systemic injection (5 mg/kg, i.p.), followed by oral administration for the subsequent 7 d via the drinking water. To assure all mice would receive the target dose of 0.1 mg/kg (Zhan et al., 2019), the water intake of each mice was measured 5 consecutive days before the CFC, and the average daily water intake was used to calculate the amount of CNO needed. During the period of CNO drinking water administration, the daily intake of each mouse was monitored by weighing the drinking bottles every morning, followed by providing fresh CNO water solution.

### Immunohistochemistry

Mice were transcardially perfused using ice-cold phosphate-buffered saline (PBS), pH 7.4, followed by ice-cold 4% paraformaldehyde (PFA) in PBS, pH 7.4. Brains were removed, postfixed overnight in 4% PFA solution, and then immersed in 30% sucrose in PBS with 0.02% NaN_3_. Brains were then sliced in 35 μm coronal sections using a cryostat and stored in PBS with 0.02% NaN_3_ at 4°C until further use. Immunohistochemical stainings were performed using standard procedures (Van den Oever et al., 2013), using the following antibodies: rat anti-Fos (1:1,000, 226017, Synaptic Systems), rabbit anti-GFP (1:1,000, A6455, Thermo Fisher Scientific), mouse anti-NeuN (1:1,000, MAB377, Merck Millipore), mouse anti-S100B (1:500, S2532, Sigma). Sections were first washed three times for 10 min in PBS and then incubated with blocking solution containing 5% normal goat serum, 2.5% bovine serum albumin, and 0.25% Triton X in PBS at room temperature for 1 h. Primary antibodies were diluted in blocking solution, and sections were incubated with primary antibody solution at 4°C overnight. Then, sections were rinsed again three times for 10 min in PBS and incubated with secondary antibodies dissolved in PBS for 2 h at room temperature. After secondary antibody incubation, slices were washed three times for 10 min in PBS, and subsequently incubated with DAPI (300 nmol/L, D3571, Thermo Fisher Scientific) in PBS solution for 15 min. Finally, slices were washed two times for 10 min in PBS and mounted using 0.2% gelatine dissolved in PBS.

Images for qualitative assessment of rM3Ds-EGFP and tdTomato expression were generated using a wide-field fluorescence microscope (Leica Microsystems, DMi8). For quantification experiments, six *z*-stacks per animal were generated using a confocal microscope (Nikon, Eclipse Ti2) with the experimenter blinded to the treatment conditions. ImageJ software was used to extract the regions of interest (ROIs) of the cells stained with DAPI (Gaussian filter, Huang threshold, watershed). Only ROIs within a predefined range for size (60–2,000 square units; to exclude nonspecific staining) and circularity (0.5–1.0) were included. To account for the fact that (parts of the) cells were often present in two or three images of a *z*-stack, MATLAB (MathWorks) was used to group the ROIs that belonged to the same DAPI^+^ cell and to subsequently count the total number of DAPI^+^ cells in a *z*-stack. Cells expressing S100B, EGFP, NeuN, tdTomato, or Fos were counted manually with the ImageJ cell counter.

### Statistical analyses

Statistical details are presented in the figure legends. Number of animals and number of cells are shown as *n*. Mice with virus misplacements were excluded from analysis (rM3Ds-EGFP = 6; EGFP = 9 for the encoding experiment: no animals were excluded for the consolidation experiment). Graphs represent mean ± standard error of the mean (SEM). GraphPad Prism software was used for statistical analysis of all data. Comparisons between and within groups were made using a two-tailed unpaired and paired Student's *t* test, respectively. When the data was not modeled by a normal distribution, it was subjected to nonparametric Mann–Whitney *U* test for between-group comparisons. In case of comparisons that involved more than two conditions, analyses were performed by two-way ANOVA followed by post hoc Bonferroni’s test. Significance was set at *p *< 0.05.

## Results

### AAV-mediated rM3Ds expression and functionality in mPFC astrocytes

**Figure 1.**
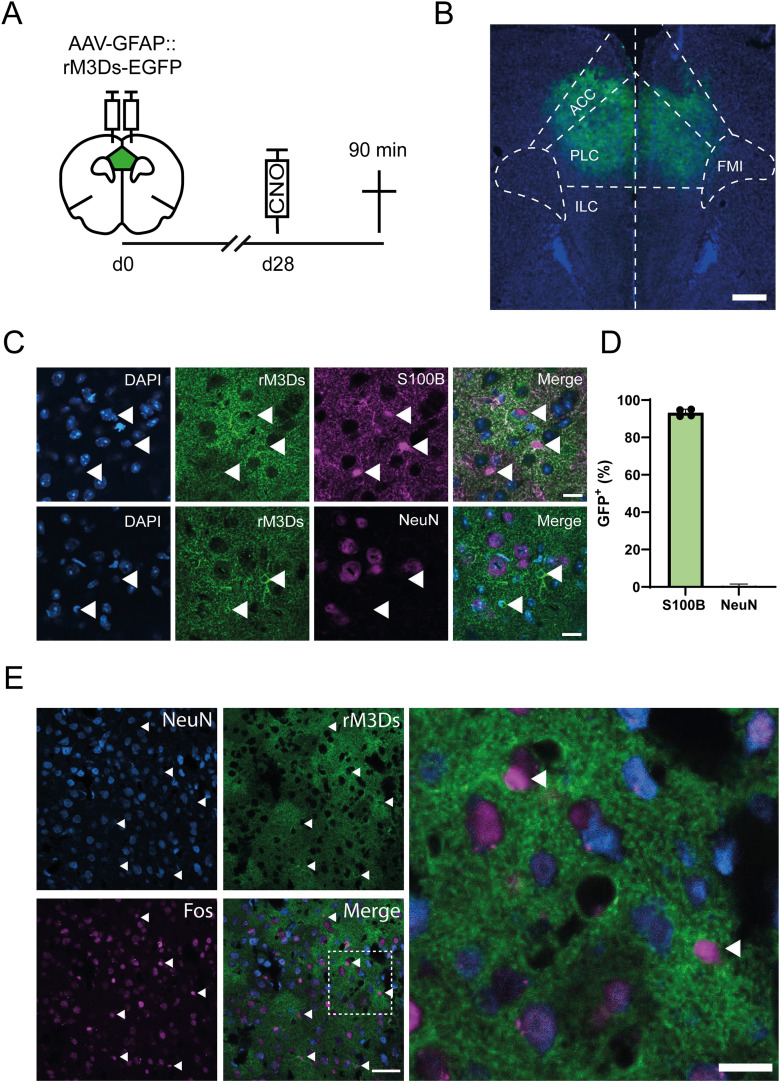
AAV-mediated rM3Ds expression and functionality in mPFC astrocytes. ***A***, Schematic timeline of rM3Ds functionality experiment. Coronal brain section indicating the mPFC region (green) wherein AAV-GFAP::rM3Ds-EGFP (*n* = 4) was injected and analyzed. CNO was injected 90 min before mice were perfused. ***B***, Representative example of AAV-GFAP::rM3Ds-EGFP expression (green) in the mPFC stained for DAPI (blue). ACC, anterior cingulate cortex; PLC, prelimbic cortex; ILC, infralimbic cortex; FMI, forceps minor of the corpus callosum. Scale bar, 500 µm. ***C***, Top row, S100B staining confirms astrocyte-specific expression of rM3Ds. Bottom row, NeuN staining demonstrates absence of rM3Ds expression in neurons. White arrowheads indicate GFP expression in cell bodies. Scale bar, 10 µm. ***D***, Quantification of colocalization of EGFP^+^/S100B^+^ cells (*n* = 4) and EGFP^+^/NeuN^+^ cells (*n* = 4). ***E***, Representative image of Fos-expressing cells. Arrowheads indicate Fos expression in EGFP^+^/NeuN^−^ cells. Left scale bar, 50 µm. Right scale bar, 10 µm.

An AAV coding for rM3Ds-EGFP, a synthetic rat M_3_ muscarinic acetylcholine receptor DREADD that is coupled to the G_s_ signaling complex (Guettier et al., 2009) fused to EGFP and under the control of a truncated variant of the glial fibrillary acidic protein (GFAP) promotor (Y. Lee et al., 2006; AAV-GFAP::rM3Ds-EGFP), was injected into the mPFC of TRAP2-tdTomato mice (Fig. 1*A*). To validate whether rM3Ds was selectively expressed in astrocytes in the mPFC (Fig. 1*B*), we quantified the colocalization of EGFP^+^ and S100B^+^ (astrocytic marker) cells and EGFP^+^ and NeuN^+^ (neuronal marker) cells (Fig. 1*C*). We observed rM3Ds expression in 93.2% ± 0.96 of S100B^+^ astrocytes (Fig. 1*D*), whereas negligible expression (0.58% ± 0.25) was found in NeuN^+^ neurons (Fig. 1*D*). To test the functionality of rM3Ds, we perfused rM3Ds-EGFP expressing mice 90 min after CNO administration (Fig. 1*A*) and stained brain sections for the immediate early gene and neuronal activity marker Fos (Cruz et al., 2015; Fig. 1*E*). Note that the 90 min timepoint corresponds to 30 min for DREADD activation (Jendryka et al., 2019) plus a delay of 60 min to visualize DREADD-induced Fos protein expression (Kovacs, 1998). Fos is typically not expressed by astrocytes under basal conditions (Cruz-Mendoza et al., 2022); however, rM3Ds-EGFP expressing astrocytes showed Fos expression after CNO treatment (Fig. 1*E*), demonstrating robust chemogenetic activation of the astrocytic G_s_ pathway.

### Activation of G_s_ signaling in mPFC astrocytes during memory encoding

**Figure 2.**
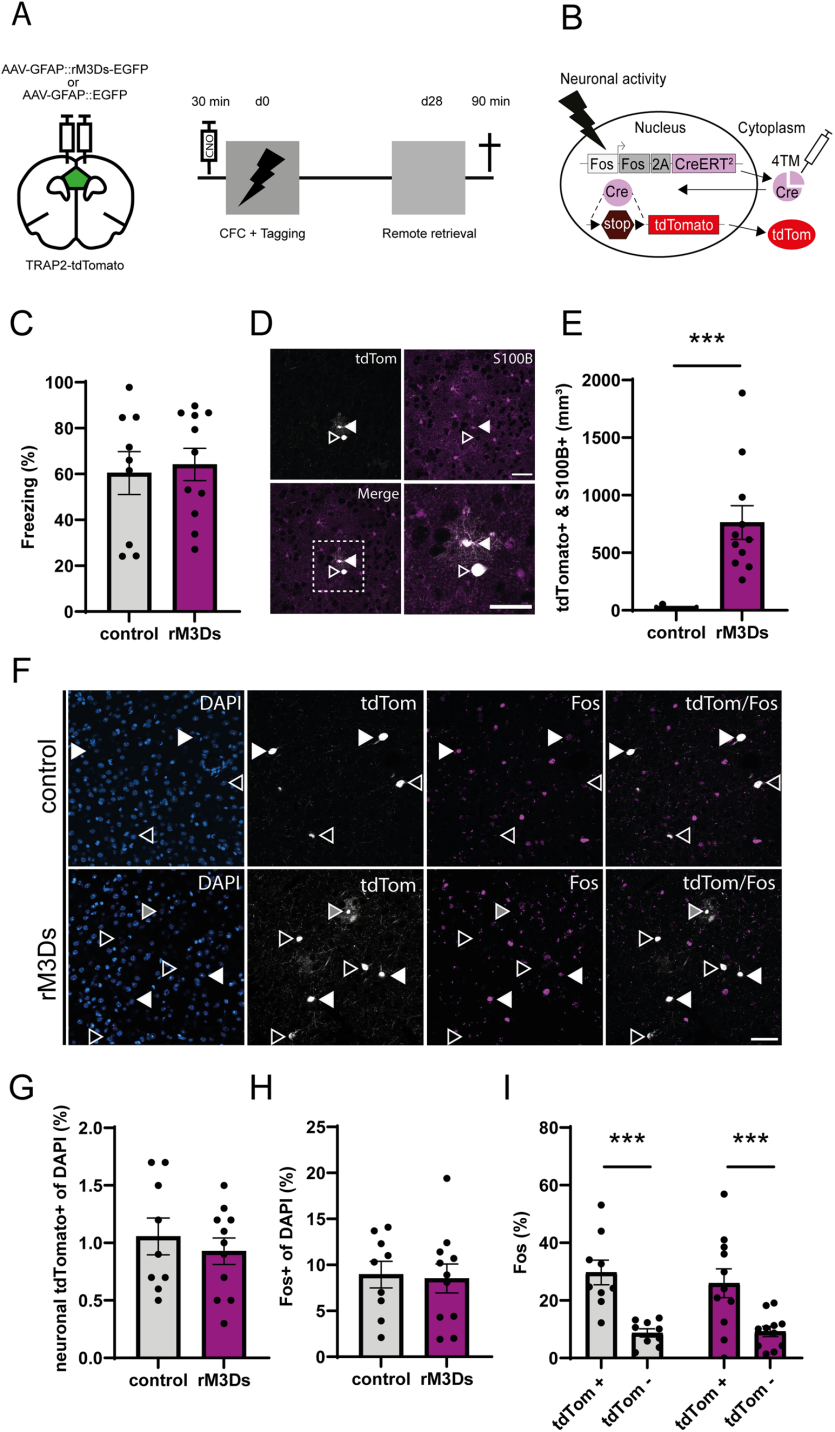
Activation of G_s_ signaling in mPFC astrocytes during memory encoding. ***A***, Schematic and timeline of the experimental design using TRAP2-tdTomato mice. CNO was injected 30 min before CFC and 4TM was injected 1 h after training to tag neurons activated during encoding with tdTomato. On Day 28 after CFC, mice underwent a remote memory test and were perfused 90 min thereafter. ***B***, Schematic of TRAP2-tdTomato labeling. Neuronal activity will induce the transcription of the Fos and CreERT2 genes indicated with the gray and lilac bars, under control of the Fos promotor shown as white bar. Only in the presence of 4TM, CreERT2 will translocate to the nucleus and remove the stop codon located between two loxP sites placed in front of the effector gene tdTomato, allowing the permanent tagging of active cells. ***C***, G_s_-DREADD activation did not affect freezing levels during remote retrieval. Unpaired *t* test: *t* = 0.32, *p* = 0.75, control (*n* = 9), rM3Ds (*n* = 11). ***D***, Example image of tdTomato-expressing cells. The white arrowhead indicates a tdTomato^+^/S100B^+^ astrocyte. The empty arrowhead indicates a tdTomato^+^/S100^−^ neuron. Scale bar, 50 µm. Extended Data Figure 2-1 shows that rM3Ds-EGFP is expressed in mPFC astrocytes, but not in neurons. ***E***, An increase in tdTomato expression was detected in astrocytes after rM3Ds activation. Mann–Whitney test: *U* = 0, ***p < 0.001, control (*n* = 9), rM3Ds (*n* = 11). ***F***, Representative images showing tdTomato^+^ and Fos^+^ cells in both groups. White arrowheads indicate reactivated (Fos^+^ in tdTomato^+^) neurons. Empty arrowheads indicate tdTomato^+^ neurons that are Fos^−^. Gray arrowheads indicate tdTomato^+^ astrocytes. Scale bar, 50 µm. Extended Data Figure 2-2*E* shows a zoom-in of Figure 2*F*. ***G***, Astrocytic G_s_ pathway activation did not alter the percentage of activated (tdTomato^+^) mPFC neurons during memory encoding. Unpaired *t* test: *t* = 0.66, *p* = 0.51, control (*n* = 9; 1.06 ± 0.16%), rM3Ds (*n* = 11; 0.93 ± 0.12%). ***H***, There was no difference in the percentage of activated (Fos^+^) neurons during the remote memory test. Unpaired *t* test: *t* = 0.2, *p* = 0.85, control 8.9 ± 1.4%; *n* = 9), rM3Ds (8.5 ± 1.6%; *n* = 11). ***I***, Percentage of Fos^+^ neurons within the tdTomato^+^ and tdTomato^−^ populations. Two-way repeated-measures ANOVA revealed a population effect: *F*_(1,18)_ = 60.33, ***p < 0.001. Post hoc Bonferroni’s test: control tdTomato^+^ versus tdTomato^−^ *p* < 0.0001; rM3Ds tdTomato^+^ versus tdTomato^−^ *p* < 0.001. Extended Data Figure 2-2 shows the number of DAPI+, tdTomato+, and Fos+ cells per cubic millimeter of all the conditions.

To determine the effect of astrocytic G_s_ pathway activation during memory encoding, while simultaneously labeling mPFC engram neurons, TRAP2-tdTomato mice expressing rM3Ds-EGFP or EGFP alone (control) in mPFC astrocytes received a systemic injection of CNO prior to CFC (Fig. 2*A*). To permanently tag and visualize neurons that were activated during CFC, all mice received 4TM 1 h after conditioning (Fig. 2*B*). Remote memory retrieval was assessed 28 d later by re-exposing mice to the conditioning context (Fig. 2*A*). We found that chemogenetic activation of the astrocytic G_s_ pathway during memory encoding did not affect freezing behavior during the remote memory test compared with control mice (Fig. 2*C*). Ninety minutes after the memory test, mice were perfused, and mPFC sections were stained for Fos (representing neurons that were activated during memory expression). Notably, in addition to tdTomato^+^ neurons, we observed tdTomato^+^ astrocytes in the rM3Ds group (762.6 ± 146 per mm^3^), whereas tdTomato^+^ astrocytes were negligible in the control group (5.844 ± 5.84 per mm^3^; Fig. 2*D*,*E*). TdTomato^+^ astrocytes were distinguishable from tdTomato^+^ neurons based on colocalization with S100B (Fig. 2*D*) and morphological hallmarks. Astrocytes had a smaller soma compared with neurons and were surrounded by elaborate cloud-like processes (Fig. 2*F*). This strongly suggests that CNO stimulated the G_s_ pathway, and thereby downstream activation of the Fos promoter and CreER^T2^ expression in mPFC astrocytes that expressed rM3Ds. Moreover, Fos expression was not observed in tdTomato^+^ (i.e., rM3Ds-expressing) astrocytes after memory retrieval, indicating that rM3Ds alone, in absence of recent CNO treatment, did not induce Fos expression in astrocytes (Extended Data Fig. 2-2*E*). As expected, rM3Ds-EGFP was not expressed in tdTomato^+^ neurons (Extended Data Fig. 2-1). The percentage of activated neurons during encoding (Fig. 2*F*,*G* and Extended Data Fig. 2-2*B*) and retrieval (Fig. 2*F*,*H* and Extended Data Fig. 2-2*C*) did not differ between groups. Next, we examined whether CFC-tagged mPFC neurons were preferentially reactivated during remote memory retrieval (Matos et al., 2019). Indeed, we found enhanced expression of Fos in the tdTomato^+^ population compared with the tdTomato^−^ population in both control and rM3Ds mice (Fig. 2*F*,*I*). However, reactivation levels did not differ between the rM3Ds and control group. Furthermore, the observed colocalization of Fos and tdTomato^+^ neurons was greater than colocalization based on chance in the control and rM3Ds groups (Extended Data Fig. 2-2*D*). The latter is in line with our observation that stimulation of astrocytic G_s_ signaling during memory encoding did not affect subsequent remote memory expression.

10.1523/ENEURO.0056-24.2024.f2-1 Figure 2-1 **Extended data for figure 2C, D. rM3Ds-EGFP expression in mPFC astrocytes, but not neurons.** Representative example of rM3Ds-EGFP expression (green) with a tdTomato tagged engram neuron (white) in the mPFC stained for DAPI (blue) and NeuN (magenta). White arrowheads indicate rM3Ds-EGFP expression in astrocytic somata and primary branches. Grey arrowheads indicate absence of rM3Ds-EGFP expression in neuronal soma and dendrite. Scale bar = 25 µm. Download Figure 2-1, TIF file.

10.1523/ENEURO.0056-24.2024.f2-2 Figure 2-2 **Extended data for figure 2F-I. Activation of G_s_ signalling in mPFC astrocytes during memory encoding. (A)** No difference was found in average number of DAPI cells per mm^3^ during activation of G_s_ signalling in mPFC astrocytes during memory encoding. Unpaired t-test: t = 0.23, p = 0.82, control (n = 9), rM3Ds (n = 11). **(B)** Astrocytic G_s_ pathway activation did not alter the percentage of activated (tdTomato^+^) mPFC neurons during memory encoding. Unpaired t-test: t = 0.70, p = 0.49, control (n = 9; 3114 ± 509.2), rM3Ds (n = 11; 2678 ± 370.2%). **(C)** There was no difference in the number of activated (Fos^+^) neurons during the remote memory test. Unpaired t-test: t = 0.26, p = 0.8, control (26218 ± 4416; n = 9), rM3Ds (24488 ± 4861; n = 11). **(D)** Percentage of observed and calculated expected overlap based on chance between the tdTomato^+^ and Fos^+^ populations after G_s_ signalling in mPFC astrocytes during memory encoding. Two-way repeated measures ANOVA revealed a significant difference between observed and chance: F(1,10) = 32.66, p < 0.001, but no interaction, nor main group effect. Post-hoc Bonferroni test: control observed vs. chance **p < 0.01; rM3Ds observed vs. chance **p < 0.01. **(E)** Representative images showing no overlap between tdTomato^+^ astrocytes and Fos^+^. Scale bar = 25 µm. Download Figure 2-2, TIF file.

### Astrocytic G_s_ pathway activation during memory consolidation

**Figure 3.**
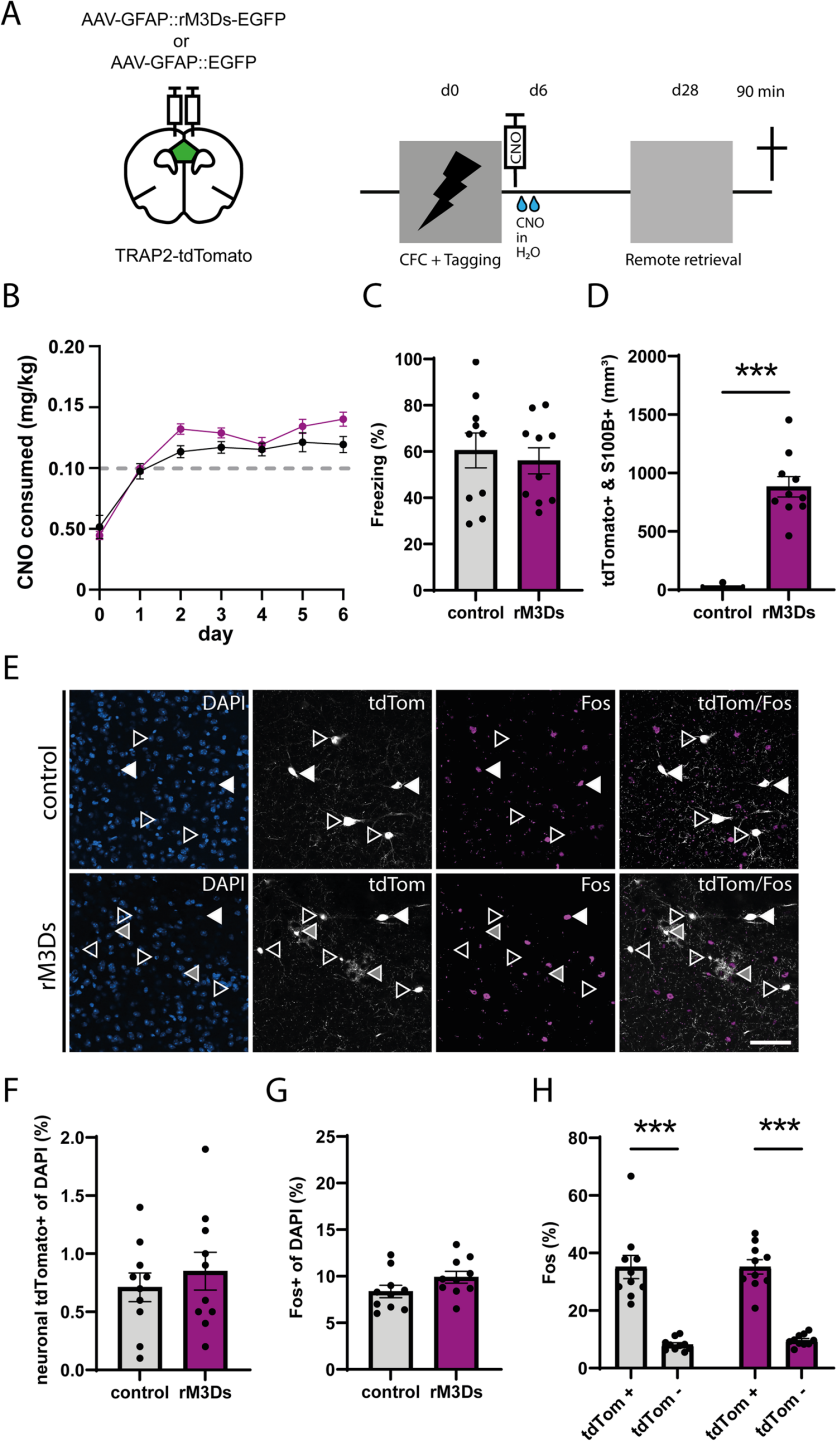
G_s_ pathway activation during memory consolidation. ***A***, Schematic and timeline of the experimental design using TRAP2-tdTomato mice. For rM3Ds activation during consolidation, CNO was first injected immediately after CFC and then administered via the drinking water until Day 7 after conditioning. 4TM was injected 1 h after training to tag neurons activated during CFC. Mice underwent a remote memory test on Day 28 after CFC and were perfused 90 min later. ***B***, Depicted is the amount of consumed CNO via drinking water, with the gray dotted line showing the target amount of 0.1 mg/kg. Two-way repeated-measures ANOVA showed no significant difference between the groups: *F*_(1,18)_ = 2.709, *p* = 0.12. ***C***, G_s_-DREADD activation during consolidation did not affect freezing during the remote memory test. Unpaired *t* test: *t* = 0.48, *p* = 0.64, control (*n* = 10), rM3Ds (*n* = 10). ***D***, CNO-mediated activation of rM3Ds induced a robust increase in tdTomato^+^ astrocytes compared with the control group. Mann–Whitney test: *U* = 0, ***p < 0.001, control (*n* = 10), rM3Ds (*n* = 10). ***E***, Representative images showing tdTomato^+^ and Fos^+^ cells in both groups. White arrowheads indicate reactivated tdTomato^+^/Fos^+^ neurons. Empty arrowheads indicate tdTomato^+^/Fos^−^ neurons. Gray arrowheads indicate tdTomato^+^ astrocytes. Scale bar, 50. ***F***, Astrocytic G_s_ pathway activation did not alter the percentage of tagged (tdTomato^+^) neurons. Unpaired *t* test: *t* = 0.69 *p* = 0.50, control (*n* = 10), rM3Ds (*n* = 10). ***G***, The percentage of activated (Fos^+^) neurons during remote memory retrieval did not differ between groups. Unpaired *t* test: *t* = 1.679, *p* = 0.11, control (*n* = 10), rM3Ds (*n* = 10). ***H***, Percentage of Fos^+^ neurons within the tdTomato^+^ and tdTomato^−^ populations. Two-way repeated-measures ANOVA revealed a significant population effect: *F*_(1,18)_ = 139.6, *p* < 0.001, but no interaction, nor main group effect. Post hoc Bonferroni’s test: control tdTomato^+^ versus tdTomato^−^ ***p < 0.001; rM3Ds tdTomato^+^ versus tdTomato^−^ ***p < 0.001. Extended Data Figure 3-1 shows the number of DAPI+, tdTomato+, and Fos+ cells per cubic millimeter of all the conditions.

To examine the effect of astrocytic G_s_ pathway activation during the first week of memory consolidation, mice expressing rM3Ds-EGFP or EGFP (control) received a systemic injection of CNO immediately after CFC and additionally received CNO via their drinking water for 7 consecutive days after conditioning. Cells that were activated during CFC were permanently labeled by 4TM injection 1 h after CFC. Remote memory retrieval was assessed 28 d later, and mice were perfused 90 min after the retrieval test (Fig. 3*A*). From Day 2 onward, the control and rM3Ds group consumed >0.1 mg/kg CNO per day (Fig. 3*B*), which has been shown to be sufficient for chemogenetic stimulation (Zhan et al., 2019). Although mice consumed <0.1 mg/kg CNO via their drinking water on Day 1, the systemic CNO injection that they received on this day ensured that rM3Ds was sufficiently stimulated. Activation of the G_s_ pathway in astrocytes during consolidation did not affect freezing behavior during subsequent remote memory retrieval (Fig. 3*C*). A low number of tagged astrocytes was observed in the control group (6.29 ± 6.29), whereas a robust increase in the number of tdTomato^+^ astrocytes was present in the rM3Ds group (881.7 ± 87.5; Fig. 3*D* and Extended Data Fig. 3-1*B*). The percentage of tdTomato^+^ neurons tagged during encoding (control 0.71 ± 0.12%; rM3Ds 0.85 ± 0.16%) and activated neurons (expressing Fos) during remote retrieval (control 8.36 ± 0.66%; rM3Ds 9.9 ± 0.64%) did not differ between groups (Fig. 3*E–G* and Extended Data Fig. 3-1*C*). Furthermore, the tdTomato^+^ population was preferentially reactivated during retrieval compared with the tdTomato^−^ population in both control (Fos^+^/tdTomato^+^ 35.15 ± 4.02%; Fos^+^/tdTomato^−^ 8.16 ± 0.67%) and rM3Ds (Fos^+^/tdTomato^+^ 35.18 ± 2.49%; Fos^+^/tdTomato^−^ 9.68 ± 0.63%) mice but did not differ between groups (Fig. 3*H*). The preferential reactivation was also significantly higher than colocalization based on chance (Extended Data Fig. 3-1*D*). Notably, tdTomato^+^ (i.e., rM3Ds-expressing) astrocytes were again not Fos positive (Extended Data Fig. 3-1*E*). Thus, the lack of differences at the cellular level is consistent with the absence of an effect of astrocytic G_s_ pathway activation during memory consolidation on remote memory expression.

10.1523/ENEURO.0056-24.2024.f3-1 Figure 3-1 **Extended data for figure 3E-H. G_s_ pathway activation during memory consolidation.** **(A)** No difference was found in average number of DAPI cells per mm^3^ during activation of G_s_ signalling in mPFC astrocytes during memory consolidation. Unpaired t-test: t = 0.13, p = 0.9, control (n = 10), rM3Ds (n = 10). **(B)** Astrocytic G_s_ pathway activation did not alter the number of tagged (tdTomato^+^) neurons. Unpaired t-test: t = 0.69 p = 0.49, control (2278 ± 435.5; n = 10), rM3Ds (2763 ± 542.8; n = 10). **(C)** The number of activated (Fos^+^) neurons during remote memory retrieval did not differ between groups. Unpaired t-test: t = 1.47, p = 0.16, control (26106 ± 2498; n = 10), rM3Ds (30781 ± 1977; n = 10). **(D)** Percentage of observed and calculated expected overlap based on chance between the tdTomato^+^ and Fos^+^ populations after G_s_ signalling in mPFC astrocytes during memory consolidation. Two-way repeated measures ANOVA revealed a significant difference between observed and chance: F(1,9) = 43.30, p = 0.0001, but no interaction, nor main group effect. Post-hoc Bonferroni test: control observed vs. chance **p < 0.02; rM3Ds observed vs. chance **p < 0.01. **(E)** Representative images showing no overlap between tdTomato^+^ astrocytes and Fos^+^. Scale bar = 25 µm. Download Figure 3-1, TIF file.

## Discussion

Our data show that chemogenetic activation of the G_s_ pathway specifically in mPFC astrocytes during memory encoding and consolidation does not alter remote contextual memory expression, nor formation and reactivation of the underlying persistent cortical engram ensemble. This suggests that increased G_s_ signaling in cortical astrocytes has no role in the formation of a persistent cortical memory engram and, together with previous published work, points to a GPCR subtype and regional specific role of astrocytic GPCRs in memory processing.

In this study, we used a single footshock CFC paradigm to ensure remote memory dependence on the persistent engram ensemble in the mPFC (Matos et al., 2019). Since G_q_- or G_i_-DREADD manipulation in the hippocampus during memory acquisition has been shown to alter fear memory expression (Adamsky et al., 2018; Kol et al., 2020), we manipulated the cortical astrocytic G_s_ pathway during CFC training, when the memory is allocated to cortical neurons, or during the first week after training, when consolidation of this newly formed engram occurs (Kitamura et al., 2017; Rao-Ruiz et al., 2021). Although we obtained null findings on the role of astrocytes in cortical memory performance, we confirmed the functionality of manipulation of G_s_ signaling in cortical astrocytes and the tagging and preferential reactivation of cortical engram neurons. Firstly, we observed robust and selective expression of the rM3Ds DREADD in the cortical astrocytes. Secondly, we showed that administration of CNO elicits the expression of Fos in rM3Ds-expressing astrocytes, which we detected as Fos protein expression and by Fos promoter-driven tdTomato expression in astrocytes of TRAP2-tdTomato mice upon chemogenetic stimulation combined with 4TM treatment. GPCR-mediated Fos expression has been reported for astrocytes in vitro, for example, the application of adrenergic receptor agonists induces Fos expression via the G_s_ pathway (Arenander et al., 1989; Cruz-Mendoza et al., 2022). In vivo GPCR-mediated Fos expression in astrocytes has been reported after G_i_- and G_q_-DREADD stimulation (Adamsky et al., 2018; Kol et al., 2020). Our data demonstrates that also strong activation of G_s_-GPCR pathways can induce Fos expression in astrocytes in vivo. Thirdly, the percentage of neurons that were tagged in the TRAP2-tdTomato mice, as visualized by tdTomato expression, did not differ between experimental and control groups and was furthermore comparable between manipulation during encoding and during consolation, confirming the reproducibility of engram cell tagging using the TRAP2-tdTomato mice. Likewise, the neurons that were active during remote retrieval, as visualized by Fos expression, did not differ between groups, and their levels were comparable between the two experiments. A relatively mild (1 footshock, 0.7 mA) conditioning paradigm allows preferential reactivation of mPFC engram neurons during remote retrieval (Matos et al., 2019). Our findings are in line with these data, as we also observed preferential reactivation of the cortical engram ensemble after remote retrieval, both in the absence or presence of astrocytic G_s_ pathway activation, during encoding and consolidation using the same conditioning protocol. Notably, ∼35% of the tagged mPFC engram ensemble was reactivated during memory retrieval in both experiments, similar to previous reports (DeNardo et al., 2019; Matos et al., 2019). Taken together, we conclude that the lack of effect of astrocytic G_s_ pathway activation on the cortical engram and remote memory expression is not due to technical errors. Instead, this manipulation seems not to alter the persistent memory engram, therefore leaving remote memory expression intact.

Previous studies reported alterations in memory function after manipulating different astrocytic GPCR subtypes (Orr et al., 2015; Adamsky et al., 2018; Kol et al., 2020). Manipulation of hippocampal astrocytes with either G_q_- or G_i_-DREADD has been shown to enhance or impair fear memory, respectively (Adamsky et al., 2018; Kol et al., 2020). These findings demonstrated the potential of astrocytic GPCR pathways in altering memory processing after CFC. To our knowledge, the role of cortical astrocytic G_s_ signaling in remote contextual fear memory had not been studied yet. A previous report showed that brain-wide ablation of the G_s_-coupled adenosine receptor A_2A_ enhances recent spatial memory retrieval, whereas stimulation of this pathway via a synthetic Rs1 receptor impairs memory consolidation (Orr et al., 2015), indicating that the astrocytic G_s_ pathway has an important role in memory processing. The discrepancy between our findings and previous studies might be explained by the use of different memory paradigms (CFC vs, e.g., Morris water maze), different methods of G_s_ pathway activation (G_s_-DREADD vs e.g., Rs1), the latter was shown to be constitutively active without its ligand (Chang et al., 2007), difference in recent versus remote memory, and/or the spatial resolution of intervention (mPFC vs brain wide/hippocampus). Importantly, since recent and remote memory processing depends on different brain regions and time scales, G_s_ pathway manipulation in astrocytes might affect recent and remote memory engrams differently. Brain-wide intervention with GPCR signaling in astrocytes may interfere with local circuitry, as well as communication between brain areas essential for memory processing, and thereby disrupt memory allocation to engram neurons and/or the consolidation and reactivation of a brain-wide memory engram network. In support of this, DREADD-mediated activation of the astrocytic G_i_ pathway in the CA1 has been shown to inhibit a hippocampal projection to the anterior cingulate cortex and thereby impair remote memory expression (Kol et al., 2020). Hence, the brain region or regions through which astrocytic G_s_ signaling affects memory processing remain to be determined, as are the different types of memories (e.g., contextual vs spatial) that this pathway may play a role in.

Our hypothesis that astrocytic G_s_ pathway activation in the mPFC would affect cortical engram function and thereby remote memory retrieval was based on the observation that G_s_-mediated elevation of cAMP levels in astrocytes, which increases glycogen breakdown and enhances lactate shuttling between astrocytes and neurons, promotes recent memory retention (Suzuki et al., 2011; Vardjan et al., 2014; Gao et al., 2016; Oe et al., 2020; Zhou et al., 2021). This was shown for G_s_ pathway activation via both endogenous and synthetic receptors. However, this evidence is primarily derived from manipulations in the hippocampus. Notably, cortical and hippocampal astrocytes are two distinct astrocytic subtypes with specific transcriptome profiles (Batiuk et al., 2020). Therefore, activation of the G_s_ pathway in the cortical astrocytes might elicit a different downstream molecular response compared with hippocampal astrocytes. This difference might explain the absence of alterations in the cortical engram ensemble and remote memory expression. In addition to possible regional differences in the downstream astrocytic response to G_s_ pathway activation, the neuronal response to activated astrocytes might also differ per brain region. Stimulation of G_s_ signaling is thought to induce morphological alterations in astrocytes (Vardjan et al., 2014), and such changes, especially in the leaflets, are known to affect synaptic function (Badia-Soteras et al., 2023). Retraction of astrocyte leaflets in hippocampal CA1 enhances fear memory, potentially via glutamate spillover from the synaptic cleft and subsequent extrasynaptic NMDAR activation (Badia-Soteras et al., 2023). Whether G_s_ pathway activation also induces astrocyte leaflet mobility in the cortex remains to be investigated, although it was recently shown that astrocyte β-adrenergic receptor activity does increase astrocyte volume and reduces extrasynaptic NMDAR recruitment, suggesting that glutamate spillover is reduced and astrocyte leaflet–synapse interaction increased (Del Franco and Newman, 2024).

To conclude, our study shows that DREADD-mediated activation of the astrocytic G_s_ pathway during fear memory encoding or consolidation does not influence the size and retrieval-associated reactivation of the mPFC engram cell population, nor remote memory retrieval. In the days to weeks after learning, cortical engrams neurons enhance and stabilize their mutual synaptic connectivity (Tonegawa et al., 2018; J. H. Lee et al., 2023), which is thought to play a key role in the maintenance and reactivation of a memory engram over time (Josselyn and Tonegawa, 2020; Rao-Ruiz et al., 2021). Therefore, we performed chemogenetic manipulation of the G_s_ pathway in cortical astrocytes during conditioning and the first week of memory consolidation. Our data suggest that G_s_ pathway activation in cortical astrocytes has no influence on the formation of a persistent contextual fear memory. It is possible that the G_s_ pathway in cortical astrocytes has a more prominent function during later stages of memory processing. Moreover, it may have a more subtle role in influencing synaptic transmission and thereby, for instance, affect the content and specificity of a memory. Gaining a better understanding of the timing and type of G_s_-, G_i_-, and G_q_-coupled GPCRs that are activated in cortical astrocytes during stages of memory processing, combined with analysis of the downstream molecular, physiological, and morphological responses, holds promise to provide important new insight into the role of astrocytes in memory persistence.

## Synthesis

Reviewing Editor: Michael Michaelides, NIDA-NIH Decisions are customarily a result of the Reviewing Editor and the peer reviewers coming together and discussing their recommendations until a consensus is reached. When revisions are invited, a fact-based synthesis statement explaining their decision and outlining what is needed to prepare a revision will be listed below. The following reviewer(s) agreed to reveal their identity: Gisella Vetere.

Reviewer 1

The authors have addressed my comments from the previous round of review and the clarity of the paper significantly improved.

Reviewer 2

I appreciate the effort of the authors addressing my concerns. Although I continuing thinking that DREADD approaches need to have a DREADD+vehicle control group instead of just EGFP alone. Also, the way to identify cell type only by DAPI-stain morphology in some of the analysis is not my more convincing way to do it. It would be support more the analysis if instead of just nuclei morphology to have added NeuN and S100 immunolabeling results for all the brain sections analyzed and no for some of them.
