# Supplementary figures and images for: Activation of Gs Signaling in Cortical Astrocytes Does Not Influence Formation of a Persistent Contextual Memory Engram
Source: eNeuro. 2024 Jun 14;11(6):ENEURO.0056-24.2024. doi: 10.1523/ENEURO.0056-24.2024 (PMC11209656; doi:10.1523/ENEURO.0056-24.2024)

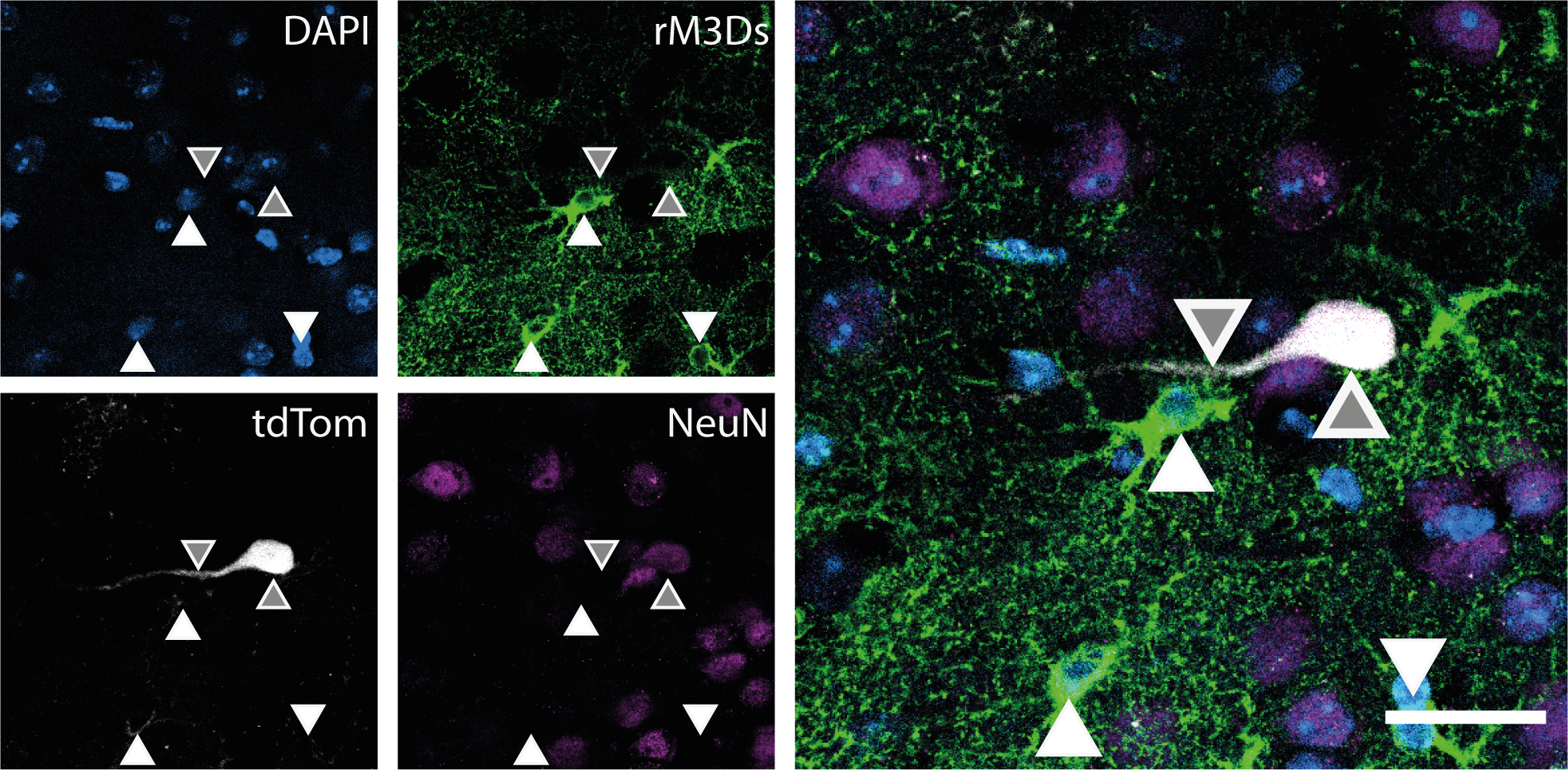

Supplement: Figure 2-1 — Extended data for figure 2C, D. rM3Ds-EGFP expression in mPFC astrocytes, but not neurons. Representative example of rM3Ds-EGFP expression (green) with a tdTomato tagged engram neuron (white) in the mPFC stained for DAPI (blue) and NeuN (magenta). White arrowheads indicate rM3Ds-EGFP expression in astrocytic somata and primary branches. Grey arrowheads indicate absence of rM3Ds-EGFP expression in neuronal soma and dendrite. Scale bar = 25 µm. Download Figure 2-1, TIF file. [file eneuro-11-ENEURO.0056-24.2024-s002.tif]

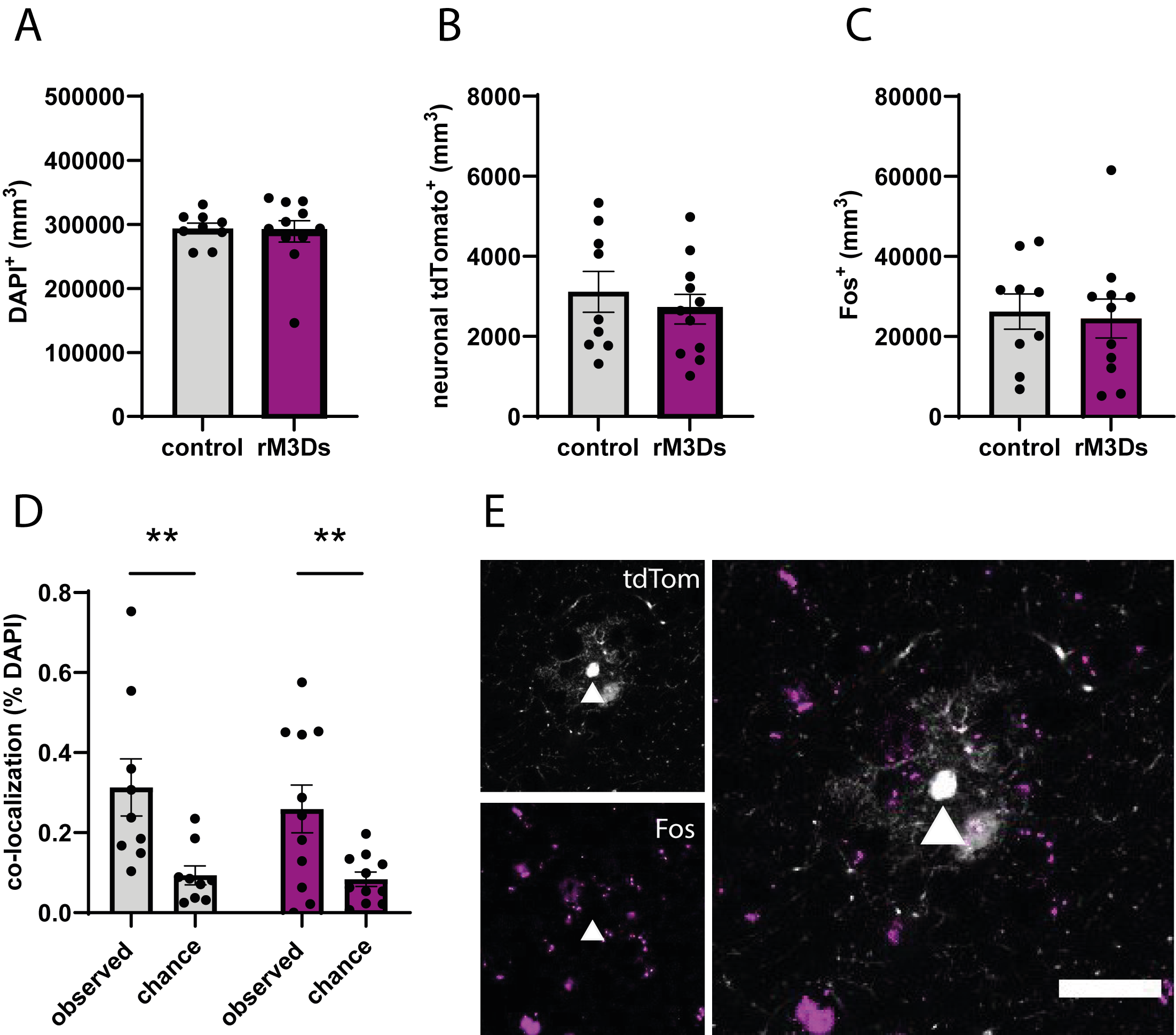

Supplement: Figure 2-2 — Extended data for figure 2F-I. Activation of Gs signalling in mPFC astrocytes during memory encoding. (A) No difference was found in average number of DAPI cells per mm3 during activation of Gs signalling in mPFC astrocytes during memory encoding. Unpaired t-test: t = 0.23, p = 0.82, control (n = 9), rM3Ds (n = 11). (B) Astrocytic Gs pathway activation did not alter the percentage of activated (tdTomato+) mPFC neurons during memory encoding. Unpaired t-test: t = 0.70, p = 0.49, control (n = 9; 3114 ± 509.2), rM3Ds (n = 11; 2678 ± 370.2%). (C) There was no difference in the number of activated (Fos+) neurons during the remote memory test. Unpaired t-test: t = 0.26, p = 0.8, control (26218 ± 4416; n = 9), rM3Ds (24488 ± 4861; n = 11). (D) Percentage of observed and calculated expected overlap based on chance between the tdTomato+ and Fos+ populations after Gs signalling in mPFC astrocytes during memory encoding. Two-way repeated measures ANOVA revealed a significant difference between observed and chance: F(1,10) = 32.66, p < 0.001, but no interaction, nor main group effect. Post-hoc Bonferroni test: control observed vs. chance **p < 0.01; rM3Ds observed vs. chance **p < 0.01. (E) Representative images showing no overlap between tdTomato+ astrocytes and Fos+. Scale bar = 25 µm. Download Figure 2-2, TIF file. [file eneuro-11-ENEURO.0056-24.2024-s003.tif]

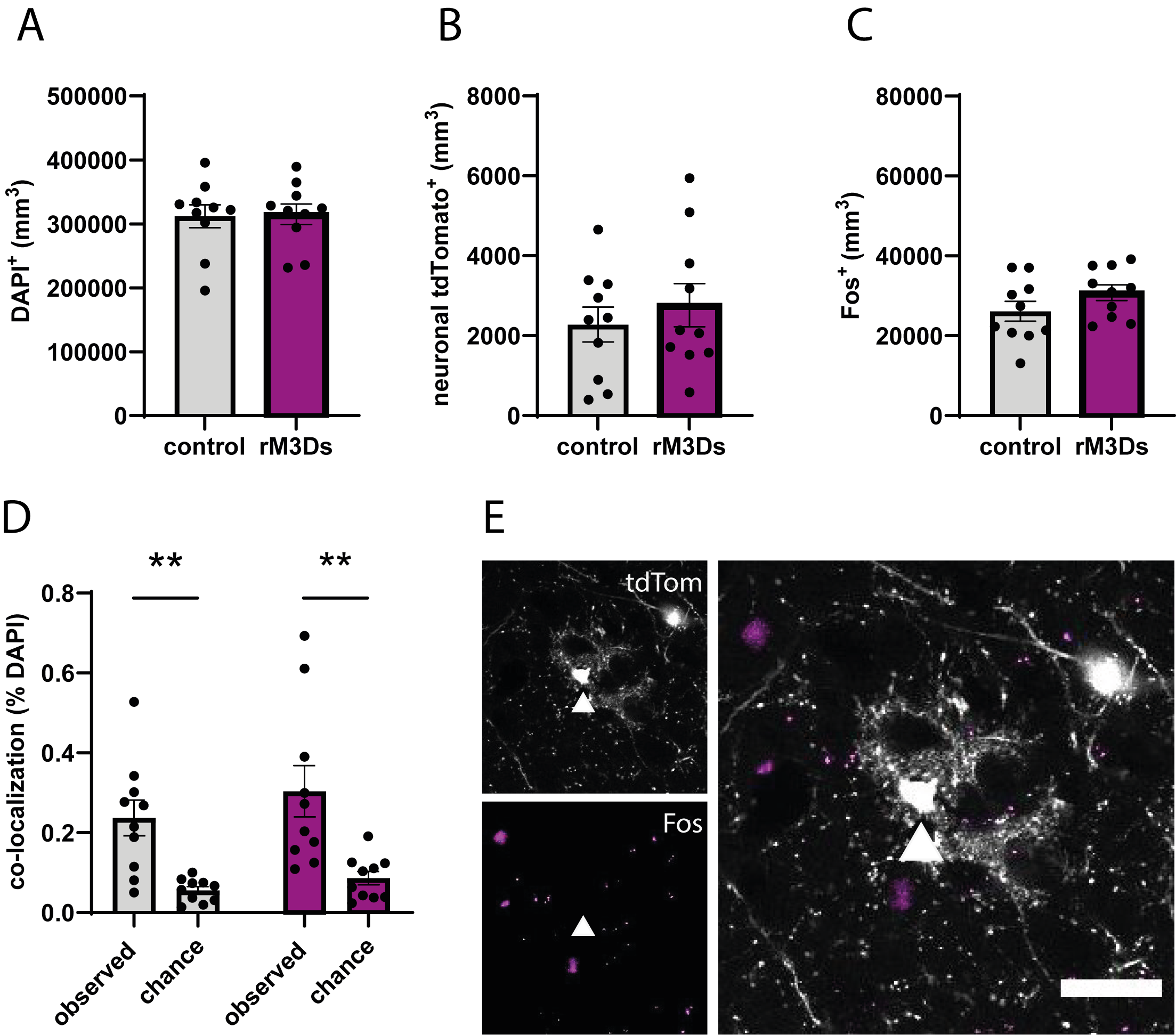

Supplement: Figure 3-1 — Extended data for figure 3E-H. Gs pathway activation during memory consolidation. (A) No difference was found in average number of DAPI cells per mm3 during activation of Gs signalling in mPFC astrocytes during memory consolidation. Unpaired t-test: t = 0.13, p = 0.9, control (n = 10), rM3Ds (n = 10). (B) Astrocytic Gs pathway activation did not alter the number of tagged (tdTomato+) neurons. Unpaired t-test: t = 0.69 p = 0.49, control (2278 ± 435.5; n = 10), rM3Ds (2763 ± 542.8; n = 10). (C) The number of activated (Fos+) neurons during remote memory retrieval did not differ between groups. Unpaired t-test: t = 1.47, p = 0.16, control (26106 ± 2498; n = 10), rM3Ds (30781 ± 1977; n = 10). (D) Percentage of observed and calculated expected overlap based on chance between the tdTomato+ and Fos+ populations after Gs signalling in mPFC astrocytes during memory consolidation. Two-way repeated measures ANOVA revealed a significant difference between observed and chance: F(1,9) = 43.30, p = 0.0001, but no interaction, nor main group effect. Post-hoc Bonferroni test: control observed vs. chance **p < 0.02; rM3Ds observed vs. chance **p < 0.01. (E) Representative images showing no overlap between tdTomato+ astrocytes and Fos+. Scale bar = 25 µm. Download Figure 3-1, TIF file. [file eneuro-11-ENEURO.0056-24.2024-s004.tif]
